# Methylation profiling reveals epigenetic programs associated with chr18 loss in small intestinal neuroendocrine tumors

**DOI:** 10.3389/fonc.2026.1885499

**Published:** 2026-10-01

**Authors:** Fredrik Axling, Samuel Backman, Per Hellman, Olov Norlén, Peter Stålberg

**Affiliations:** Department of Surgical Sciences, Uppsala University, Uppsala, Sweden

**Keywords:** chromosome 18 loss, DNA methylation, epigenetics, long-read sequencing, small intestinal neuroendocrine tumors

## Abstract

**Introduction:**

Small intestinal neuroendocrine tumors (SI-NETs) are characterized by a low mutational burden and limited recurrent driver mutations, suggesting that other mechanisms contribute to tumor development. One of the most frequent genomic alterations in SI-NETs is loss of heterozygosity on chromosome 18 (chr18del), which defines a major molecular subtype. However, the epigenetic consequences of this event remain poorly understood.

**Methods:**

In this study, we used long-read whole-genome sequencing to characterize genome-wide DNA methylation patterns in twelve primary tumors. Native methylation detection and copy-number profiling enabled stratification of tumors according to chromosome 18 status and direct comparison of epigenetic states between chr18del and non-chr18del (diploid) tumors.

**Results:**

Chr18del tumors displayed widespread hypomethylation relative to diploid tumors, while chromosome 18 showed spatially structured regional methylation differences, including localized regions of increased methylation. Cross-platform validation using an independent Illumina EPIC methylation cohort confirmed the direction and distribution of many methylation events. Integration with the corresponding transcriptomic dataset revealed that a subset of methylation changes was associated with differential gene expression consistent with established promoter- and gene-body methylation models. Functional categorization of these methylation states revealed regulatory patterns linked to neuronal signaling, intracellular signaling cascades, and interactions with the extracellular matrix.

**Discussion:**

Together, these findings support an association between chromosome 18 status and widespread methylation differences that extend beyond the affected chromosome and are linked to transcriptional programs relevant to neuroendocrine tumor biology.

## Introduction

Small intestinal neuroendocrine tumors (SI-NETs) constitute a clinically significant category of neoplasms, characterized by heterogeneous biological features, ranging from slow-growing tumors to disease presenting with metastatic lesions. Although advances in diagnostic imaging and systemic therapies have been made, the application of precision-based treatment approaches is still restricted, and delayed diagnosis is frequently observed, leading to unfavorable clinical outcomes (1–3).

Genomic investigations using array-based platforms and short-read sequencing have revealed that SI-NETs possess a low mutational burden relative to other solid tumors (4). Recurrent coding driver mutations are infrequent, and the majority of reported alterations are observed at low frequencies across different patient cohorts. While mutations impacting SMAD-related signaling pathways and other genes have been documented, the only consistently recurring mutation is found in CDKN1B, which is present in only about 8% of cases (5–7).

These observations imply that mechanisms beyond recurrent point mutations may be more important during tumorigenesis. Increasing evidence suggests that epigenetic dysregulation, particularly aberrant DNA methylation, could be a significant source of molecular alterations in these tumors (8–10).

The most frequently observed event across cohorts is the loss of heterozygosity on chromosome 18 (chr18del), which is present in roughly 50-70% of tumors (11–14). Chr18del may manifest as an initial and isolated genomic alteration; in contrast, tumors without this event commonly display chromosomal gains, deletions, and genomic instability (15, 16). Clinically, chr18del tumors have been correlated with more favorable outcomes compared to non-chr18del (diploid) tumors, demonstrating improved progression-free survival, which aligns with a less aggressive disease (9, 17). Together, these findings indicate that chromosome 18 status represents a major axis of molecular stratification in SI-NETs.

The molecular pathways connecting the loss of chromosome 18 to specific tumor subtypes are not yet fully elucidated, despite the frequent occurrence. The observed differences in molecular patterns may not be accounted for by chromosomal deletions alone, implying a multifaceted interplay between chromosomal loss, epigenetic control, and gene expression (18).

Given that chr18del constitutes a larger chromosomal alteration rather than a localized mutation, molecular associations with chromosome 18 status may extend beyond genes located on chromosome 18 and involve broader genome-wide regulatory differences. Long-read genome-wide DNA methylation sequencing enables the simultaneous assessment of DNA methylation and copy-number variation (CNV), allowing direct comparison of chr18del and diploid tumors and facilitating the identification of epigenetic patterns associated with chromosome 18 status.

## Materials and methods

### Ethics statement

Written informed consent for research use of tissue was obtained from all patients. Ethical approval was granted by the Regional Ethical Review Board in Uppsala (EPN 2011/375; approval date 2016-07-13), and all procedures were conducted in accordance with the Declaration of Helsinki.

### Patient samples and nanopore sequencing

Twelve frozen primary ileal SI-NET tumor specimens were obtained from our biobank. Tumor cell content was verified by hematoxylin-eosin staining. Patient-matched normal samples were collected from non-tumor tissue, including intestine or liver, or from peripheral blood. These samples were sequenced as part of the broader long-read whole-genome workflow and primarily served as matched germline reference material for somatic SNV analyses. They were not included in the differential methylation analyses reported here.

High-molecular-weight DNA was extracted using the Monarch HMW DNA Extraction Kit (New England Biolabs, Ipswich, Massachusetts, USA). DNA concentration and integrity were assessed using NanoDrop 2000 (Thermo Scientific, Waltham, Massachusetts, USA), Qubit dsDNA assays (Invitrogen, Waltham, Massachusetts, USA), and the FEMTO Pulse system (Agilent, Santa Clara, California, USA).

Sequencing libraries were prepared using the SQK-LSK114 ligation kit (Oxford Nanopore Technologies, Oxford, United Kingdom) and sequenced on PromethION R10.4.1 flow cells for 72 hours, using one flow cell per sample. Basecalling was performed in super-accurate mode using Dorado (Oxford Nanopore Technologies, Oxford, United Kingdom) with a minimum quality score Q ≥ 10. The resulting unmapped BAM (uBAM) files were used for downstream analyses.

### Read alignment and methylation calling

Sequencing reads (uBAM) were aligned to the GRCh38 reference genome (GCA_000001405.15) using Dorado (Oxford Nanopore Technologies, Oxford, United Kingdom) and processed with Samtools (19) for sorting and indexing. CpG methylation was detected using Modkit (Oxford Nanopore Technologies, Oxford, United Kingdom) with pileup and the following filters: mapping quality ≥ 20, minimum pileup depth ≥ 10, CpG context only, and a fixed modification-call probability threshold of ≥ 0.7 applied consistently across all samples. Sample-specific CpG methylation tables were generated for downstream methylation analyses.

### Copy number analysis

Copy-number profiles were inferred from aligned long-read sequencing data to determine chromosome 18 status. Copy-number segmentation was performed using QDNAseq (20). Tumors were classified as chr18del or diploid based on copy-number alterations and allelic imbalance across chromosome 18.

### Long-read differential methylation analysis

Differential methylation analysis was performed using the DSS R package (21, 22). Differentially methylated loci (DML) were identified using the DMLtest function with smoothing enabled and followed by callDML. Loci were retained if the absolute methylation difference was ≥ 0.1 and the false discovery rate (FDR) ≤ 0.05. Differentially methylated regions (DMRs) were identified using callDMR on the DMLtest output, with a methylation difference threshold ≥ 0.1, *p*-value ≤ 0.05, and a minimum of three CpGs per region. Only autosomal loci and regions were retained.

To assess whether chromosome 18-associated methylation differences could be influenced by reduced sequencing coverage resulting from chromosome 18 loss, a sensitivity analysis was performed. For each tumor, median chromosome 18 coverage was compared between chr18del and diploid groups using a Wilcoxon rank-sum test. Relative chromosome 18 coverage was calculated as the median chromosome 18 coverage divided by the median coverage across the remaining autosomes. Chromosome-wide weighted methylation was calculated as the total number of modified calls divided by the sum of modified and canonical calls. Chromosome-wide weighted methylation estimates were additionally recalculated after restricting CpGs to minimum coverage thresholds of 10X, 15X, 20X, and 25X.

### EPIC array processing

Illumina Infinium Methylation EPIC array data (18) were processed using the minfi package (23) with Noob normalization (24). Samples were retained if more than 95% of probes had detection *p*-values < 0.02. Probes failing detection, located on sex chromosomes, overlapping SNPs, or previously reported as cross-reactive (25) were excluded. Only high-quality autosomal probes were retained.

Using GRCh38 genomic coordinates, probes were ordered by genomic position and grouped into adaptive genomic windows defined by clusters of probes located within 10 kb. Windows containing fewer than three probes were excluded. Mean β-values were calculated separately for chr18del and diploid tumors, and Δβ (chr18del – diploid) values were derived. Windows were retained if ≥ 70% of probes showed concordant methylation direction. High-confidence windows were defined as those with Δβ ≥ 0.10.

### Adaptive window-based integration of nanopore and EPIC methylation signals

DML were mapped to EPIC windows using genomic overlap and overlapping DML were aggregated to estimate the mean long-read methylation difference per window. DMRs were evaluated using fractional overlap, defined as the proportion of each DMR’s genomic length intersecting one or more EPIC-supported windows. DMRs were considered EPIC-supported when ≥ 20% of their length overlapped EPIC windows.

EPIC methylation differences were quantified per window as the difference in mean β-values between groups. Directional concordance between platforms was assessed by comparing the sign of EPIC and long-read methylation differences within overlapping windows. Quantitative agreement between EPIC and Nanopore signals was evaluated using Pearson correlation and linear regression across matched windows.

### Annotation and permutation analysis

EPIC-supported DMRs were annotated relative to gene-body and promoter regions using UCSC GRCh38 gene models. Promoters were defined as -2 kb to +200 bp relative to transcription start sites (TSS). Additional annotations included CpG context (island, shore, shelf, open sea), ENCODE (26) candidate cis-regulatory elements (cCREs), and FANTOM5 (27) enhancers.

Statistical significance of cross-platform overlap was assessed using permutation testing (1,000 iterations). EPIC windows were randomly redistributed within an EPIC-accessible genomic universe defined by 2-kb tiles containing at least one EPIC probe. Overlap significance was quantified using area-weighted DMR statistics to generate empirical *p*-values.

### RNA-seq processing and differential expression analysis

RNA-seq data (18, 28) were processed using a unified workflow. Reads were quality-trimmed (Phred score ≥ 30) (29) and quantified using Salmon (30) in quasi-mapping mode against a genome-decoy-aware GRCh38 transcriptome. Transcript abundances were summarized to gene-level expression values.

For the Alvarez dataset, chromosome 18 copy-number status was inferred using RNAseqCNV (31), integrating gene-level read depth using FeatureCounts (32) and SNV-derived allelic imbalance based on GATK RNA-seq variant calling (33). Samples were classified as chr18del or diploid based on arm-level copy-number status: those exhibiting loss of 18p alone, 18q alone, or both 18p and 18q were designated as chr18del, whereas samples with a 0/0 status were classified as diploid.

Differential expression between chr18del and diploid tumors was assessed using Wilcoxon rank-sum tests. Only genes with ≥ 50 counts, as determined by the smallest comparison group (63 samples in Patte et al. and 19 samples in Alvarez et al.), were retained to ensure sufficient detection sensitivity and to enable reliable downstream validation using PCR-based approaches. *P*-values were corrected using the Benjamini-Hochberg procedure, and genes with FDR < 0.05 were considered significant. Gene Ontology (GO) enrichment analysis was performed separately for genes assigned to the *Stable*, *Unstable*, and *Repressed* methylation-defined states using g:Profiler (34). The statistical background of the enrichment analysis consisted of genes passing the expression and methylation filtering criteria and therefore eligible for methylation-expression classification.

### Single-cell RNA-seq pseudobulk validation

Single-cell RNA-seq datasets from three independent SI-NET studies (35–37) were obtained from NCBI GEO and the Federated European Genome-phenome Archive (FEGA). Data were processed using Seurat (38), including quality filtering, normalization, dimensionality reduction, clustering, and UMAP visualization.

Tumor neuroendocrine cell clusters were identified using canonical markers reported in the respective studies. Pseudobulk profiles were generated by summing raw counts across tumor cells for each sample. Counts were log2-transformed, and batch effects between studies were corrected using the limma package (39) prior to downstream validation analyses.

## Results

Nanopore long-read whole-genome sequencing was performed on twelve primary SI-NET tumors with matched normal samples collected at surgical resection and stored in our biobank. Copy-number profiles derived from the same sequencing data stratified tumors into chr18del and diploid groups, forming the basis for subtype-specific methylation analyses. Native 5mC methylation was directly detected from the long-read data, enabling genome-wide CpG profiling without bisulfite conversion. Tumor samples achieved a mean coverage of 28.5X (SD 5.1X) with a median read N50 of 18.0 kb (IQR 15.9-22.2 kb) (**Supplementary Material 1**).

To validate long-read methylation signals and account for potential platform-specific biases, an independent cohort of 147 primary SI-NET tumors (18) profiled with the Illumina Infinium Methylation EPIC array was analyzed and integrated using an adaptive window approach to identify regions with concordant methylation direction (**Figure 1**). Chromosome 18 status in this cohort was determined using the published copy-number annotations derived from short-read whole-genome sequencing, enabling parallel stratified methylation analyses across platforms (**Supplementary Material 2**).

**Figure 1:**
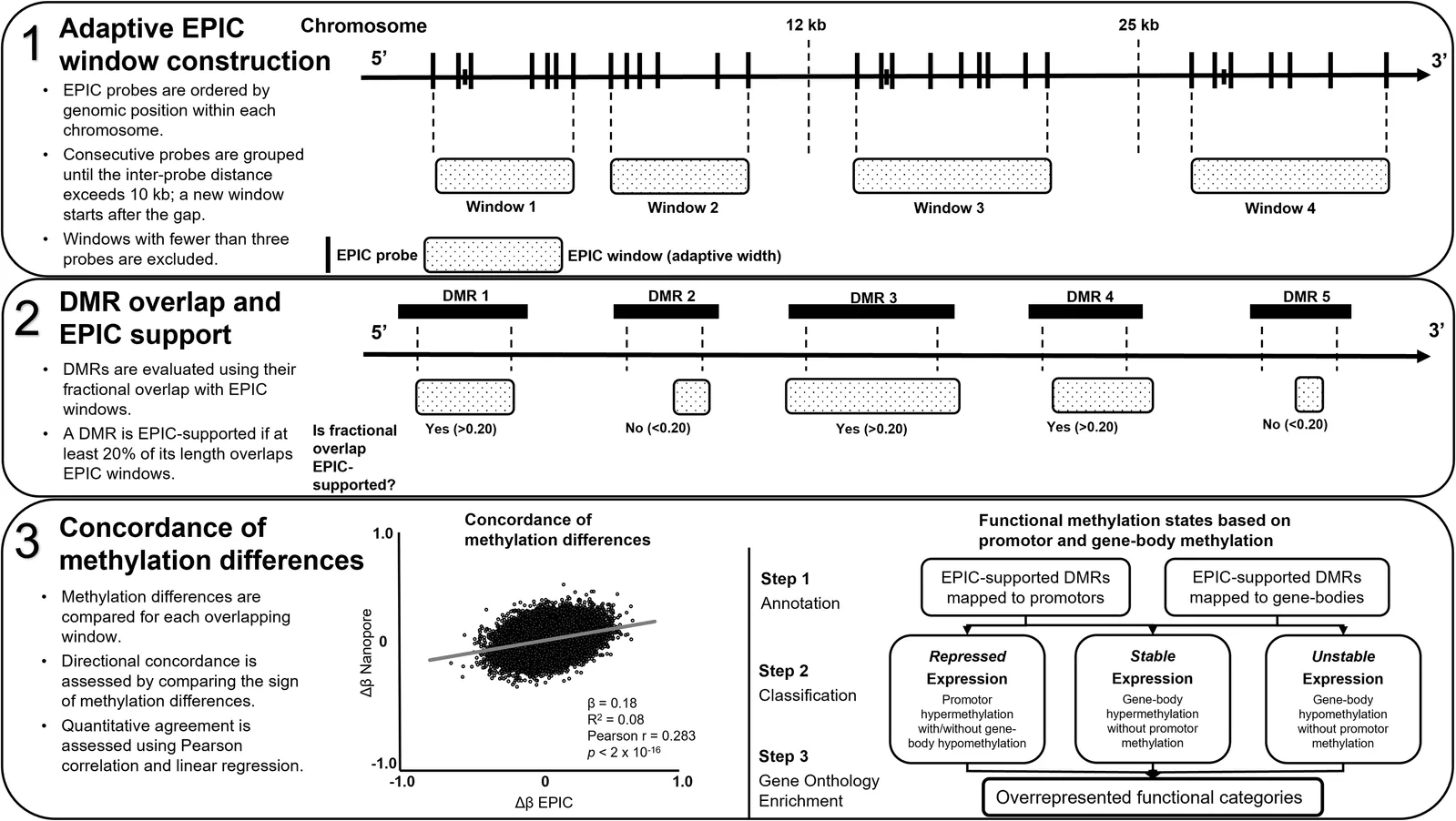
Overview of cross-platform validation of differential DNA methylation using Nanopore sequencing and EPIC arrays. (1) EPIC probes are grouped into adaptive genomic windows based on probe density. (2) Nanopore differentially methylated regions (DMRs) are evaluated for EPIC support based on fractional overlap with EPIC windows. (3) Concordance between platforms is assessed by comparing Δβ values, directional agreement, and Pearson correlation. EPIC-supported DMRs are annotated to promoters or gene bodies, classified into functional methylation states (Repressed, Stable, or Unstable), assessed for gene expression, and analyzed for Gene Ontology enrichment.

### Genome-wide and regional differential methylation

Genome-wide Nanopore methylation profiling identified ~4.2 million differentially methylated loci (DML) between chr18del and diploid tumors. Among these, ~3.0 million loci (71.3%) were hypermethylated in diploid tumors and ~1.2 million (28.7%) in chr18del tumors. Mean methylation differences were +0.166 and -0.168, respectively, indicating broadly balanced but directionally opposing genome-wide methylation changes between the two tumor groups.

At the regional level, 82796 differentially methylated regions (DMRs) were detected. Of these, 55542 regions (67.1%) were hypermethylated in diploid tumors and 27254 (32.9%) in chr18del tumors. Regional methylation differences (+0.216 and -0.224) exceeded the single-locus effect sizes described above, consistent with coordinated methylation shifts across clustered CpG sites.

### Chromosomal distribution of chr18del-associated methylation changes

Genome-wide comparison of methylation differences between chr18del and diploid tumors revealed consistent directional patterns across platforms. Mean Δβ values were predominantly negative, indicating global hypomethylation in chr18del tumors compared with diploid tumors. Chromosome 18 displayed pronounced, spatially structured regional methylation differences, including regions with increased methylation in chr18del tumors. At the chromosome-average level, however, support for the chromosome 18-specific methylation effect was limited across platforms. Nanopore sequencing showed a positive mean methylation difference on chromosome 18, whereas the corresponding EPIC estimate was closer to zero. In contrast, genome-wide regional methylation differences showed high directional concordance between platforms (88.4%). Thus, the chromosome 18-specific effect was not strongly corroborated by EPIC at the chromosome-average level, although regional cross-platform concordance was high. In contrast, chromosomes 9, 19, and 22, among others, exhibited marked hypomethylation in chr18del tumors (**Figure 2**).

**Figure 2:**
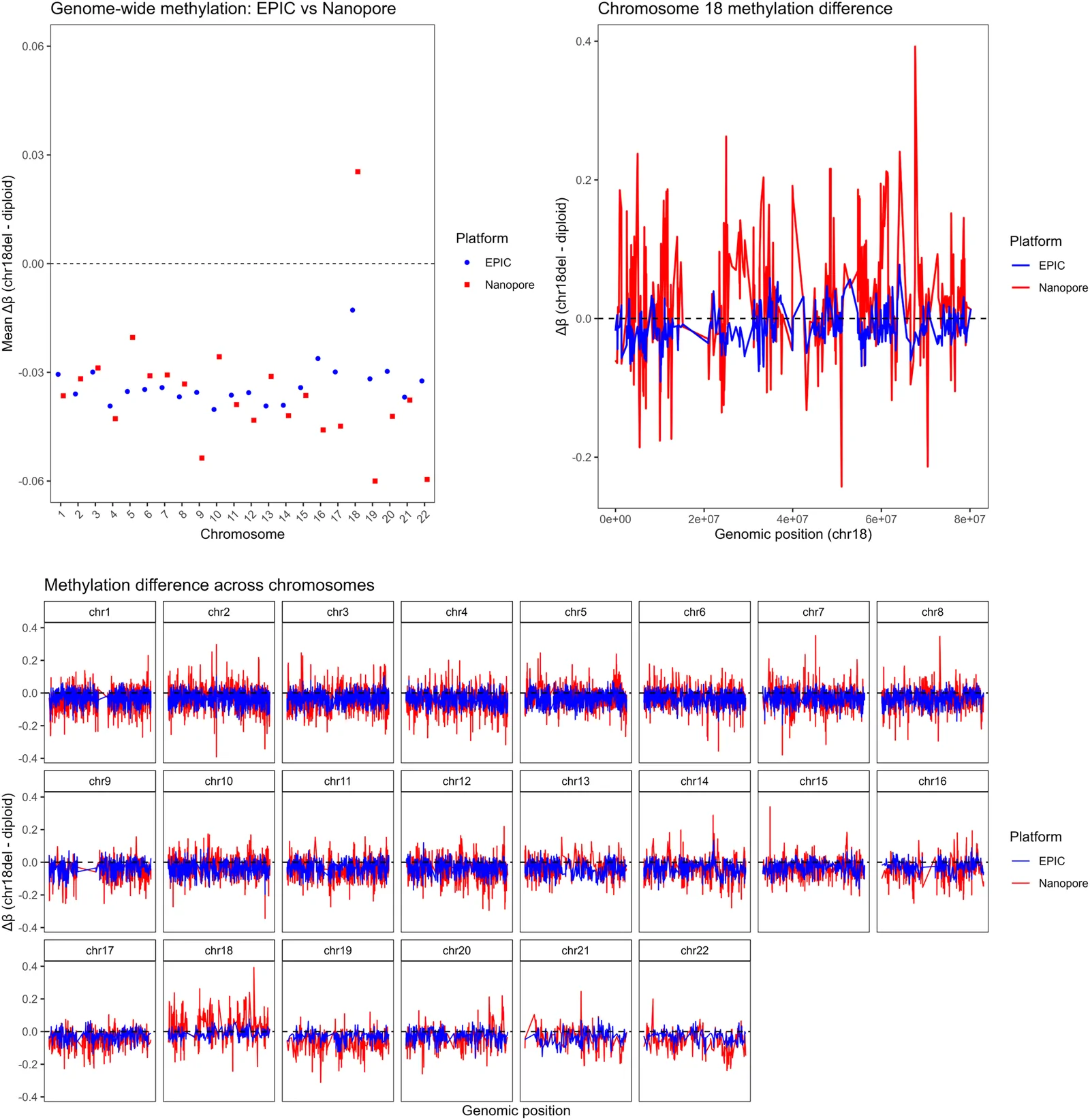
Cross-platform comparison of genome-wide methylation differences between Nanopore sequencing and EPIC arrays. Top left: Average chromosome-level methylation differences (Δβ chr18del - diploid) for Nanopore (red) and EPIC (blue). Top right: Adaptive-window Δβ profile across chromosome 18. Bottom: Genome-wide adaptive-window Δβ profiles across chromosomes 1-22, with platform-specific signals overlaid, Nanopore (red), EPIC (blue). Each panel represents one chromosome.

To assess whether reduced sequencing coverage could account for the chromosome 18-associated methylation signal, we performed a sensitivity analysis. Chr18del tumors showed reduced chromosome 18 sequencing coverage compared with diploid tumors. Median chromosome 18 coverage was 13X in chr18del tumors and 27X in diploid tumors (*p* = 0.013). Relative chromosome 18 coverage, normalized to coverage across the remaining autosomes, was likewise reduced in chr18del tumors (median 0.62 versus 1.04; *p* = 0.0057). In contrast, chromosome-wide weighted methylation levels were similar between groups (median 0.730 versus 0.734; p = 0.745), and methylation estimates remained stable across minimum coverage thresholds of 10X to 25X (**Supplementary Material 3**).

### Cross-platform validation of differential methylation using EPIC arrays

Following preprocessing and quality control of the EPIC dataset, 79.8% of probes were retained for analysis. Differential methylation analysis identified 276905 significant probes (FDR < 0.05), of which 48587 (17.5%) showed methylation differences of Δβ ≥ 0.10. To enable regional comparison with Nanopore-derived signals, EPIC probes were aggregated into adaptive genomic windows defined by clusters of at least three probes within 10 kb that displayed coherent methylation directionality. The adaptive-window strategy maintains biologically coherent CpG groupings while avoiding arbitrary boundaries imposed by fixed-width binning.

This procedure yielded 18,613 coherent EPIC windows, of which 857 met both coherence and effect-size criteria and were classified as strong windows. For cross-platform comparison, Nanopore-derived DMRs were mapped against the full set of coherent EPIC windows rather than the strong subset. Of the 82,796 Nanopore DMRs, 18,664 (22.5%) overlapped at least one of the 18,613 coherent EPIC windows (**Table 1**).

**Table 1:** Cohort characteristics and differential methylation summary across Nanopore and EPIC platforms.

A. Study and platform characteristics					
Study	Platform	Tumor	Chr18del	Diploid	Coverage
Axling et al.	Nanopore long-read WGS (5mC)	12	7 (58.3%)	5	Genome-wide CpG coverage
Patte et al.	Illumina EPIC array	147	63 (42.8%)	84	Probe-based (~850k loci)
Locus-level differential methylation					
Category	Number of Loci				
Nanopore DML	4200080				
EPIC significant probes (FDR < 0.05)	276905				
EPIC probes (Δβ ≥ 0.10)	48587				
Regional differential methylation					
Category	Number of Regions				
Nanopore DMRs	82796				
EPIC coherent windows (≥3 probes, ≥70% coherence)	18613				
EPIC strong windows (Δβ ≥ 0.10)	857				

The table summarizes locus- and region-level differential methylation across platforms, including Nanopore DML/DMRs and EPIC probe and window signals after coherence and effect-size filtering.

Permutation analysis using 1,000 randomized EPIC window sets demonstrated that this overlap exceeded random expectation (empirical p < 0.001). General directional concordance between platforms was high (88.4%; exact binomial test p < 2 × 10^-16^; 95% CI 0.879-0.888). This concordance reflects overlapping regional methylation signals and does not imply equivalent agreement in chromosome-wide average methylation, particularly for chromosome 18. Regression analysis further demonstrated a significant association between Nanopore and EPIC methylation differences (β = 0.18, R^2^ = 0.08, p < 2 × 10^-16^; Pearson r = 0.283), supporting concordant directional methylation changes within shared genomic regions across platforms (**Supplementary Material 4**).

Nanopore sequencing detected methylation changes with greater amplitude, whereas EPIC arrays exhibited a more compressed dynamic range, reflecting inherent technological limitations. Nevertheless, methylation shifts showed consistent patterns of genomic distribution across platforms, and downstream analyses focused on differentially methylated regions supported by the EPIC dataset.

### Gene body and promoter differential methylation

EPIC-supported DMRs were annotated and stratified by genomic context, distinguishing gene-body and promoter-associated intervals. Consistent with the earlier genome-wide analysis, chr18del tumors exhibited overall hypomethylation relative to diploid tumors, whereas chromosome 18 displayed localized hypermethylation (**Figure 3**, top).

**Figure 3:**
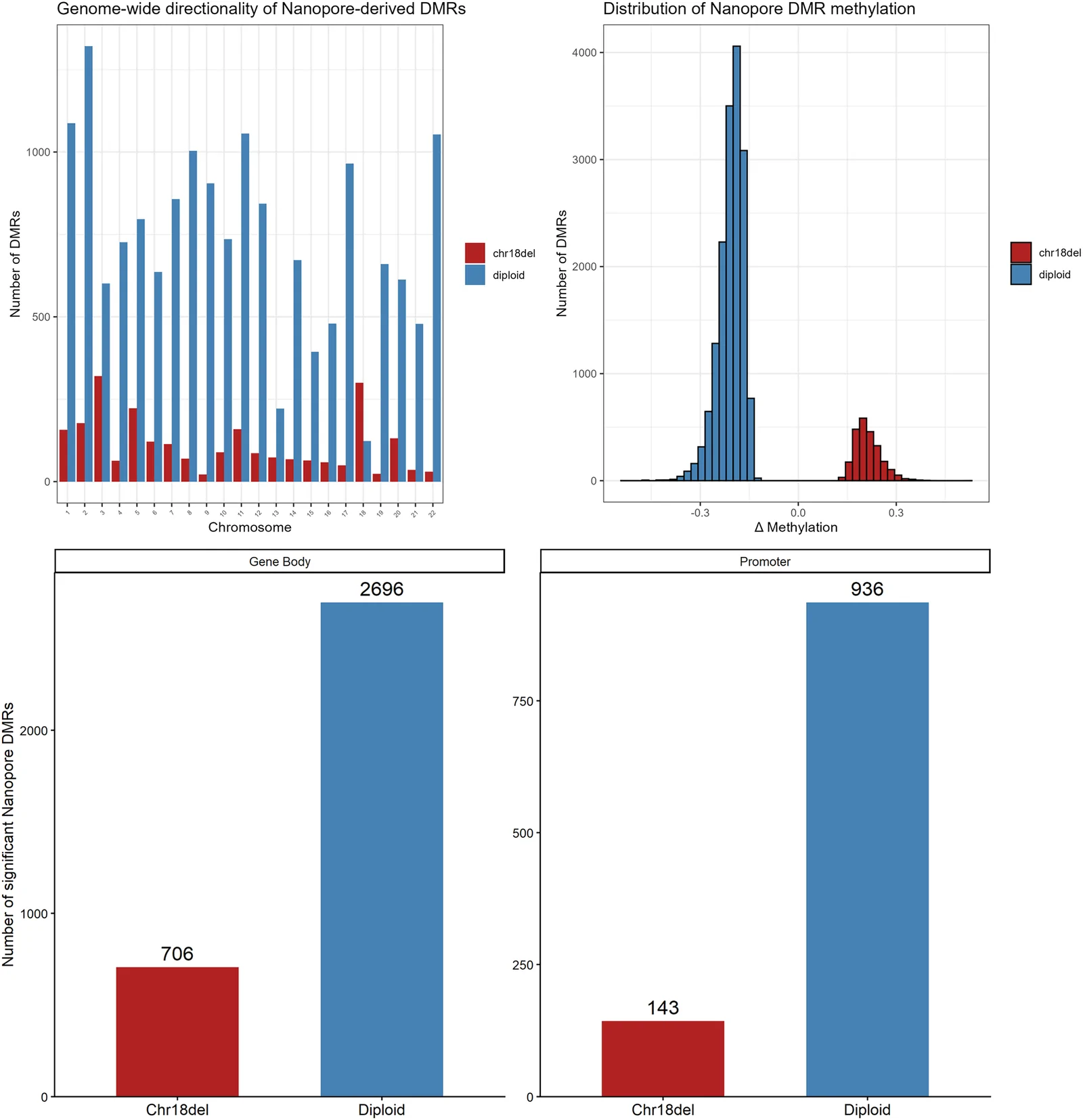
Genome-wide distribution and directionality of EPIC-validated Nanopore-derived differentially methylated regions (DMRs) in SI-NET. Top left: Chromosomal distribution of significant DMRs between chr18del and diploid tumors. Top right: Distribution of Δ methylation values across all significant DMRs. Bottom: Number of significant DMRs located in gene bodies and promoters stratified by chr18del and diploid groups. Methylation direction is indicated by color: chr18del-associated hypermethylation (red) and diploid-associated hypermethylation (blue).

Among EPIC-supported DMRs uniquely mapped to gene bodies (*n* = 3402), 706 were hypermethylated in chr18del tumors and 2696 in diploid tumors, indicating a predominance of diploid-associated gene-body hypermethylation. Promoter-associated regions showed a similar distribution. Of 1079 EPIC-supported promoter DMRs, 143 were hypermethylated in chr18del tumors and 936 in diploid tumors (**Figure 3**, bottom).

Together, these results indicate that cross-platform validated methylation differences occur within both gene bodies and promoter regions and are predominantly driven by increased methylation in diploid tumors.

### Cross-cohort investigation of chr18del-associated transcriptional changes

The EPIC methylation cohort reported by Patte et al. included gene expression data for the majority of samples, enabling integration of promoter and gene-body methylation profiles with corresponding transcriptional output within the same cohort. Differentially expressed genes identified in Patte et al. were then compared with the independent Alvarez et al. dataset (28) to assess the reproducibility of chr18del-associated expression patterns. Given its smaller sample size and lower statistical power, the Alvarez dataset was used primarily for cross-cohort validation of candidate expression patterns rather than for functional enrichment analysis.

In the Patte et al. cohort (*n* = 147), chromosome 18 status (42.8% chr18del) was defined using short-read whole-genome sequencing. In contrast, the Alvarez et al. cohort (*n* = 44) inferred chr18del status (43.2%) directly from RNA-seq profiles. Differential expression analysis comparing chr18del and diploid tumors (FDR < 0.05) identified 4,916 significant genes in the Patte cohort and 425 in the Alvarez cohort. Of these, 349 genes were shared between datasets (7.0% of Patte; 82.1% of Alvarez), whereas 76 genes were unique to the Alvarez cohort and 4567 were unique to the Patte cohort.

Among the overlapping genes, directional concordance was high: 47.3% showed concordant downregulation and 52.1% concordant upregulation in chr18del tumors across both cohorts. Only a small fraction of the overlapping genes (n = 2) displayed discordant regulation between datasets. Despite the smaller Alvarez cohort and consequently reduced statistical power, 349 genes were significant in both datasets and showed nearly complete directional agreement, supporting a subset of chromosome 18 status-associated expression differences that were consistent across cohorts (**Figure 4**).

**Figure 4:**
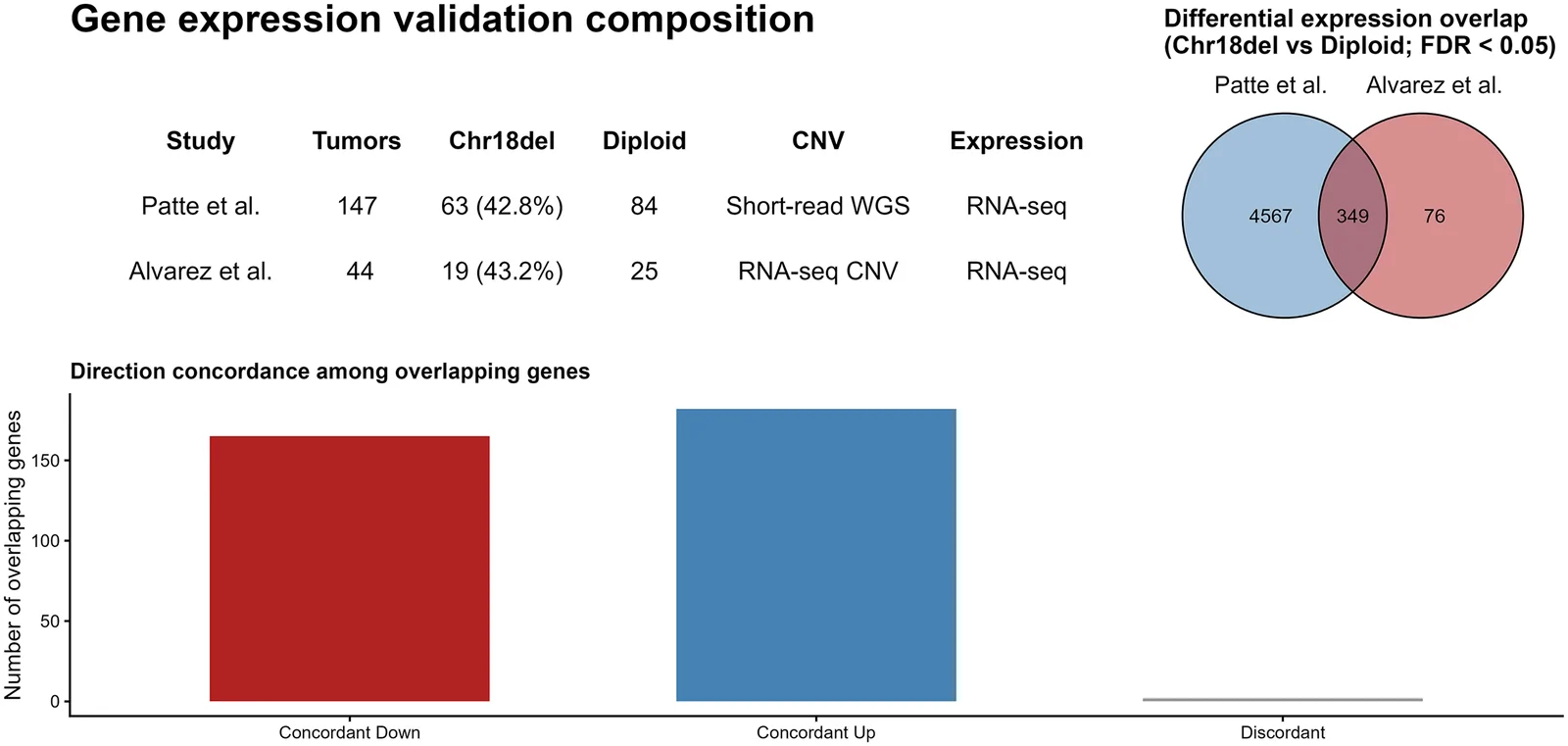
Cross-cohort chr18del-associated differential expression in SI-NET. Top left: Study design and cohort composition for the RNA-seq datasets used for validation. Top right: Overlap of significantly differentially expressed genes (FDR < 0.05) between chr18del and diploid tumors in the Patte et al. and Alvarez et al. cohorts. Bottom: Directional concordance of expression changes among the overlapping genes.

### Directional methylation-expression relationships in chr18del tumors

Given the availability of matched methylation and gene expression data in the Patte cohort, we examined whether methylation differences between chr18del and diploid tumors were accompanied by concordant transcriptional changes consistent with established epigenetic models. Methylation-expression relationships are often non-linear and context-dependent, depending on CpG density, genomic location, regulatory architecture, and the number of affected CpG sites.

Promoter and gene-body methylation states were evaluated to examine their relationship with gene expression in chr18del tumors. To focus on robust associations, effect-size thresholds were applied, retaining genes with significant differential expression (FDR < 0.05), an absolute log_2_ fold change ≥ 0.5, and a methylation difference ≥ 0.15. Among genes within this filtered set, 232 gene-body hypermethylation events were associated with increased expression, whereas 138 gene-body hypomethylation events corresponded to decreased expression. In contrast, promoter hypermethylation was predominantly linked to reduced expression, occurring in 53 genes, while only a single gene exhibited promoter hypomethylation with increased expression.

### Functional methylation state classification

Based on the established relationships of promoter and gene-body methylation, genes were grouped into three functional methylation states. Genes exhibiting promoter hypermethylation, with or without accompanying gene-body hypomethylation, were classified as *Repressed*, consistent with promoter-associated transcriptional silencing. Genes with gene-body hypermethylation in the absence of promoter methylation were categorized as *Stable*, reflecting patterns associated with productive transcriptional elongation. In contrast, genes with gene-body hypomethylation without promoter methylation were classified as *Unstable*, consistent with reduced transcriptional stability.

Applying this framework identified 87 *Stable* genes that were upregulated in chr18del tumors, whereas 54 *Unstable* genes and 44 *Repressed* genes exhibited decreased expression (**Supplementary Material 5**).

### Functional pathway enrichment of methylation-defined gene sets

To explore the biological processes associated with the methylation-defined transcriptional states, the 185 genes defining the three functional states were subsequently analyzed for functional enrichment. The Gene Ontology enrichment analysis was performed after excluding genes located on chromosome 18. Genes assigned to the *Stable* state were enriched for neuronal and synaptic compartments, whereas genes in the *Unstable* state were enriched for cellular signaling, communication, and membrane-associated processes. The *Repressed* state showed enrichment for extracellular-region and extracellular-space annotations supported by multiple genes, together with sulfurtransferase- and cyanate-related terms driven by *MPST* and *TST*; the latter should therefore be interpreted cautiously (**Figure 5**). The relative gene distribution and expression direction of the three methylation-defined states are summarized in **Figure 6** (left).

**Figure 5:**
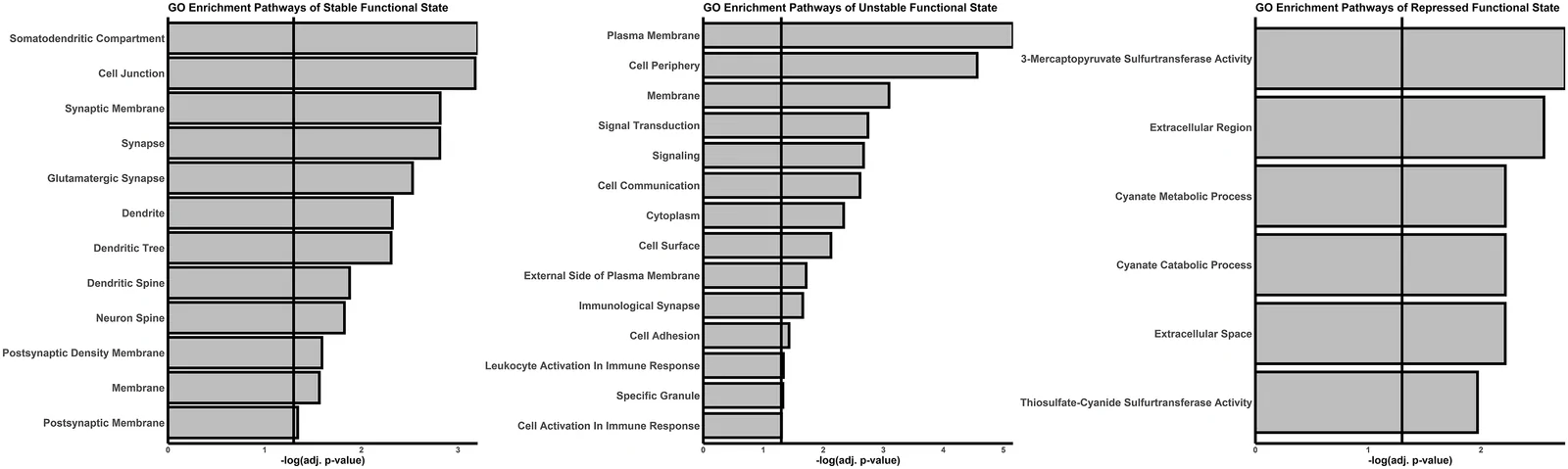
Gene Ontology (GO) enrichment of functional states derived from Patte et al. GO enrichment analysis of genes associated with the Stable (left), Unstable (middle), and Repressed (right) functional states. Bars represent significantly enriched GO terms ranked by -log10 (adjusted p-value). The vertical line indicates the significance threshold (adjusted p = 0.05).

**Figure 6:**
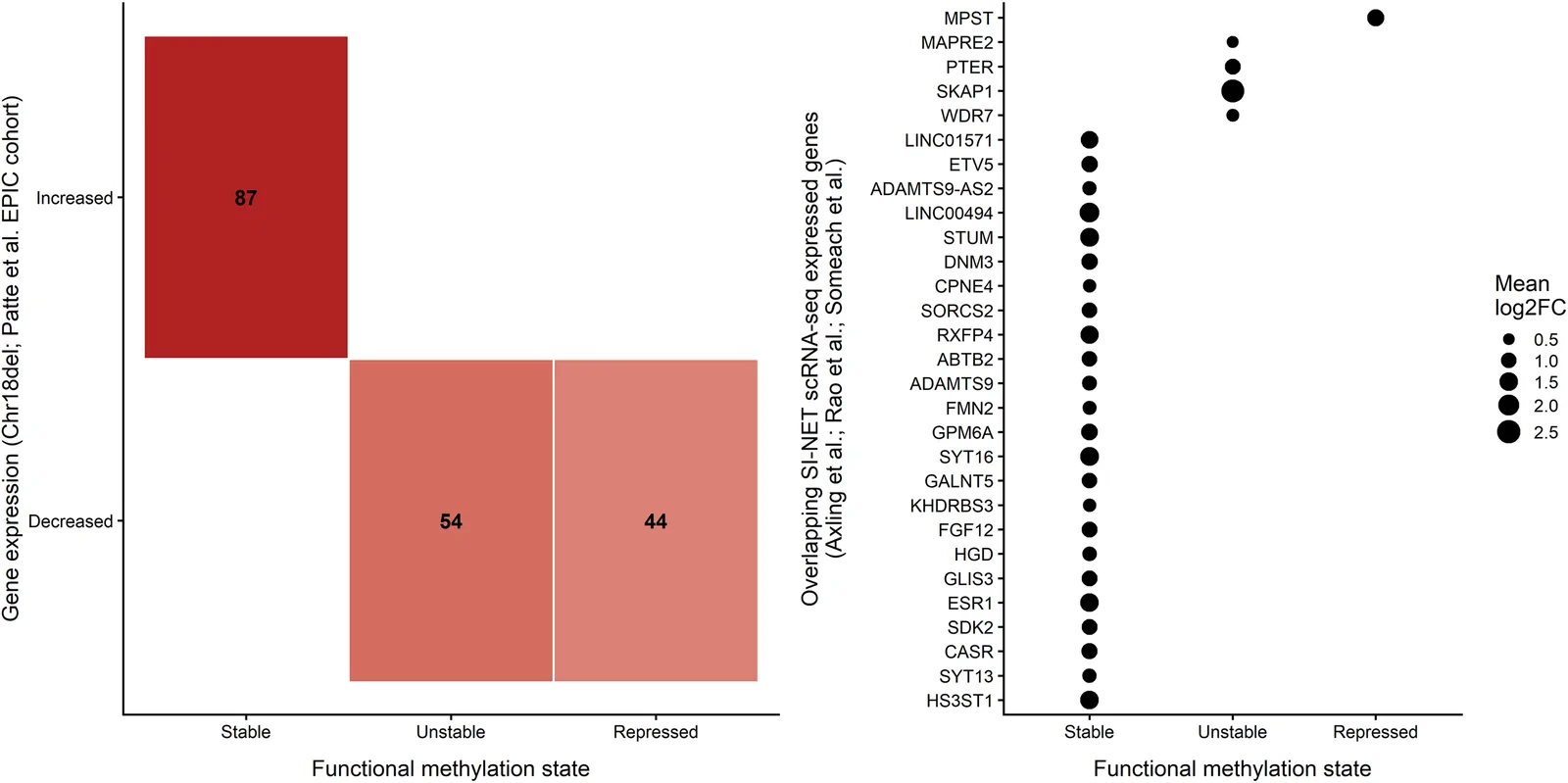
Candidate genes and tumor-cell expression in SI-NET. Left: Distribution of number of candidate genes assigned to the Stable, Unstable, and Repressed methylation-defined states according to their associated expression direction in chr18del tumors. Numbers indicate the number of genes in each state. Right: Expression patterns of the 30 cross-cohort methylation-defined candidate genes in the Patte et al. and Alvarez et al. datasets. Candidate genes were additionally evaluated in three independent single-cell RNA-seq datasets to confirm detectable expression in SI-NET tumor cells and reduce the likelihood that bulk-tissue signals were primarily attributable to non-tumor cell populations. Point size represents mean absolute log_2_ fold change across the Patte et al. and Alvarez et al. cohorts.

The ten genes within the methylation-defined set mapped to chromosome 18 and were all classified as *Unstable*. As reduced expression of genes located on the deleted chromosome may reflect direct gene-dosage effects, these genes were excluded from the Gene Ontology enrichment analysis and were not used for epigenetic interpretation. Functional enrichment therefore reflects genes located outside chromosome 18 (**Supplementary Material 6**).

### Cross-cohort validation for methylation-defined candidate genes

To assess cross-cohort reproducibility of methylation-defined gene sets and identify candidates for further functional studies, their expression was evaluated in the Alvarez et al. gene-expression dataset. This analysis retained a total of 30 genes: 25 genes (28.7%) from the *Stable* state, compared with 4 genes (7.4%) from the *Unstable* state and 1 gene (2.2%) from the *Repressed* state, indicating that the *Stable* transcriptional program displayed the highest cross-cohort reproducibility. Among the cross-cohort *Stable* candidates, *ADAMTS9*, *ADAMTS9-AS2*, *CASR*, and *SYT13* were of particular interest, showing increased expression in chr18del tumors together with methylation patterns consistent with the *Stable* state.

### Tumor-cell validation using single-cell transcriptomic datasets

To assess whether the 30 methylation-defined candidate genes were expressed by SI-NET tumor cells rather than predominantly reflecting non-tumor cell populations in bulk tissue, their expression was examined in single-cell RNA-seq datasets from three independent SI-NET studies (35–37). All but one gene (*CIBAR1-DT*) was detected in SI-NET tumor cells across the three datasets, supporting tumor-cell expression of the candidate set (**Figure 6**, right).

## Discussion

In this study, we used long-read whole-genome sequencing to characterize the DNA methylation landscape of small intestinal neuroendocrine tumors (SI-NETs). By the combination of native methylation detection with simultaneous copy-number profiling, tumors were classified based on chromosome 18 status, reflecting the most common genomic alteration in SI-NETs, which has already been correlated with detectable epigenetic and transcriptional outcomes (15–18). Tumors with chr18del showed a genome-wide predominance of hypomethylated DMRs compared with diploid tumors. These differences were regionally heterogeneous rather than uniformly distributed across entire chromosomes.

Broader copy-number architecture represents a potential source of heterogeneity in SI-NETs, as CNA-defined subgroups characterized by chromosome 18 loss and recurrent chromosomal gains have been described in previous studies (9, 17, 18). Additional CNVs were present in several tumors in the present long-read cohort, including recurrent gains involving chromosomes 4, 5, 7, 10, 14, and 20. However, the limited sample size precluded meaningful statistical stratification according to multi-CNV status. We therefore retained chromosome 18 status as the primary grouping variable and did not attempt to determine the independent contribution of other copy-number alterations to the observed methylation patterns.

Integration with the independent dataset from Patte et al. (18) provided cross-platform support for a subset of the methylation differences identified by Nanopore sequencing. Because the EPIC array provides more limited CpG coverage, probes were aggregated into adaptive regional windows rather than compared by direct probe overlap. Approximately 22.5% of Nanopore-derived DMRs overlapped EPIC-derived windows and showed consistent directional effects. This concordance primarily reflects agreement at the regional level rather than equivalent chromosome-wide average methylation differences across platforms. Consistent with this, chr18del tumors were also associated with hypomethylated groups in the clustering analysis performed by Patte et al., while localized regions with increased methylation were observed within the broader hypomethylation pattern. Together, these findings support regionally structured methylation differences associated with chromosome 18 status rather than uniform genome-wide shifts, consistent with the observations of Patte et al.

Integration with the Patte expression dataset, followed by candidate validation using the Alvarez dataset, showed that a subset of regional methylation differences was associated with differential gene expression between chr18del and diploid tumors. These relationships were evaluated so that functional interpretation was based on both methylation location and transcriptional direction rather than on hyper- or hypomethylation alone.

The resulting methylation patterns were classified according to established promoter- and gene-body methylation frameworks, in which promoter methylation is generally associated with transcriptional repression, whereas gene-body methylation can be associated with transcriptionally active states (40–42).

Only a limited subset of methylation-associated genes mapped to chromosome 18. For these cis-associated genes, reduced expression may reflect direct gene-dosage effects and therefore cannot be attributed independently to gene methylation. The principal functional interpretation was consequently based on trans-associated genes located outside chromosome 18, where methylation and transcriptional differences were associated with several regulatory and biological pathways.

Functional enrichment analyses suggested that the methylation-defined gene states were associated with biological processes relevant to SI-NET biology, including neurosecretory function, intracellular signaling, and interactions with the extracellular matrix. These processes are consistent with the functional characteristics of enterochromaffin cells, which depend on specialized secretory mechanisms and interactions with their microenvironment.

Several candidate genes showing associated methylation and expression patterns were consistently found to be upregulated and classified as *Stable* in the chr18del group. These genes include the extracellular matrix metallopeptidase *ADAMTS9* and its antisense transcript *ADAMTS9-AS2*; both have been identified as epigenetically regulated tumor suppressors (43–46). These findings raise the possibility that altered expression of these genes may relate to extracellular matrix remodeling and tumor-stroma interactions within the SI-NET microenvironment. Other genes associated with neuroendocrine signaling and secretory function were also associated with the *Stable* functional state in chr18del tumors, such as the calcium-sensing receptor (*CASR*), which has been reported to be epigenetically regulated in gastroenteropancreatic neuroendocrine tumors (47), and the vesicle trafficking regulator Synaptotagmin 13 (*SYT13*), whose expression is consistent with altered neurosecretory machinery in enterochromaffin-derived tumor cells (48, 49).

The detection of tumor-cell expression of these candidates was further supported by three independent single-cell SI-NET datasets (35–37), reducing the likelihood that the observed bulk-tissue expression patterns were primarily driven by non-tumor cell populations. The limited replication of the *Unstable* and *Repressed* states nevertheless indicates that these categories require further evaluation in additional SI-NET expression datasets.

While certain expression patterns were consistent across cohorts, substantial quantitative expression differences were observed between the Patte and Alvarez datasets (18, 28). These discrepancies likely reflect methodological and cohort-specific factors, including differences in cohort size, sequencing design, library depth, and chromosome 18 status inference. The Alvarez cohort was smaller and generated using single-end, unstranded RNA sequencing, whereas the Patte cohort used paired-end, stranded sequencing. Salmon quantification also showed higher median library depth in Patte et al., with 48.5 million processed and 42.0 million mapped fragments per sample, compared with 33.4 million and 28.1 million, respectively, in Alvarez, which may have reduced transcript-detection sensitivity and statistical power in the latter dataset. In addition, chromosome 18 status was determined using whole-genome sequencing in Patte but inferred from RNA-seq in Alvarez.

Several limitations warrant consideration. The Nanopore cohort was relatively small, reflecting the present cost and logistical challenges of long-read whole-genome sequencing of clinical tumor samples. Although validation in a larger EPIC methylation cohort and independent transcriptomic datasets provides external support for several observed patterns, these datasets do not fully compensate for the limited statistical power of the twelve-sample long-read discovery cohort. Additionally, as the methylation calls were not resolved by haplotype, allele-specific methylation cannot be excluded as a contributor to chromosome 18-localized methylation differences. The relatively permissive, fixed modification-probability threshold of 0.7 was selected to increase signal capture in the Nanopore discovery cohort, with independent EPIC validation used to prioritize reproducible regional signals; however, this threshold may retain a greater proportion of lower-confidence modification calls and should be considered when interpreting Nanopore-specific findings. Larger long-read cohorts will therefore be required to establish the generalizability of chromosome 18 status-associated methylation patterns, clarify their underlying mechanisms, and assess the extent to which additional CNAs could change these associations.

Finally, the present analysis primarily evaluates methylation changes through their association with transcriptomic output and may therefore miss regulatory mechanisms not reflected in the transcriptome. Future studies integrating chromatin accessibility and other regulatory-layer data will be needed to further characterize the mechanisms underlying chromosome 18 status-associated epigenetic differences.

## Data Availability

Publicly available datasets used in this study included the Patte et al. EPIC methylation dataset (NCBI GEO: GSE229203) and transcriptomic dataset (EGA: EGAD50000000906), the Alvarez et al. RNA-seq dataset (NCBI GEO: GSE98894), and single-cell RNA-seq datasets from Rao et al. (NCBI GEO: GSE140312), Somech et al. (NCBI GEO: GSE292163), and Axling et al. (Federated EGA Sweden [FEGA]: EGAD50000002265).The long-read sequencing data generated in the present study, including tumor samples and matched normal samples, have been deposited in FEGA under accession EGAD50000002975. Access to controlled EGA and FEGA datasets is governed by the respective repository data-access procedures.
